# A Case of Mucinous Adenocarcinoma in the Setting of Chronic Colitis Associated With Intestinal Spirochetosis and Intestinal Stricture

**DOI:** 10.1097/MD.0000000000000493

**Published:** 2015-01-30

**Authors:** Shintaro Akiyama, Daisuke Kikuchi, Toshifumi Mitani, Takeshi Fujii, Akihiro Yamada, Akira Matsui, Osamu Ogawa, Toshiro Iizuka, Shu Hoteya, Mitsuru Kaise

**Affiliations:** From the Department of Gastroenterology (SA, DK, TM, AY, AM, OO, TI, SH, MK); and Department of Pathology (TF), Toranomon Hospital, Tokyo, Japan.

## Abstract

Mucinous adenocarcinoma (MC) is a unique pathological type of colorectal cancer (CRC). The development of MC is often associated with intestinal inflammation and/or microsatellite instability (MSI). Moreover, MC has clinicopathological characteristics that render making the correct diagnosis difficult such as extramural progression. Meanwhile, intestinal spirochetosis (IS) is a condition in which colonic epithelial cells are colonized and/or infected by spirochetes. Intestinal inflammation due to IS occurs by the destruction of colonic microvilli and induces chronic diarrhea. Recently, it was reported that the prevalence of IS tended to be high in patients with sessile serrated adenomas/polyps, the precursor of MSI-high CRC including MC.

This study presents a case of MC in the setting of intestinal inflammation due to IS and tries to clarify the cause of MC development by performing immunohistochemical stain of resected specimen for DNA mismatch repair (MMR) proteins.

This patient is a 63-year-old man with no symptoms who had a positive fecal occult blood test. Subsequent endoscopic findings and biopsy results revealed intestinal stricture of the transverse colon and chronic infective colitis associated with IS. Metronidazole therapy was initiated but was not effective. Although follow-up colonoscopy was performed repeatedly, intestinal perforation occurred 20 months later. Subtotal colectomy and ileostomy were performed. Pathological examination of resected specimens revealed MC with normal expression of MMR proteins, including MLH1, MSH2, MSH6, and PMS2. The histopathological classification was Union for International Cancer Control (UICC) IIIB and adjuvant chemotherapy was initiated.

This is an interesting case of MC developing in the setting of chronic colitis associated with IS. It seemed that the cause of MC development was not MSI but intestinal inflammation. Besides, endoscopic diagnosis of MC in this case was difficult because of the extramural progression and lack of obvious atypical colonic glands in biopsy specimens. This report provides evidence for an association between neoplasm and IS-induced intestinal inflammation. Moreover, we suggest that making the diagnosis of MC could be difficult because of its unique clinicopathological characteristics.

## INTRODUCTION

Mucinous adenocarcinoma (MC) has different clinicopathological and molecular characteristics. First of all, MC arises in the setting of intestinal inflammation, including inflammatory bowel disease (IBD) and irradiation-associated colitis.^1–3^ However, microsatellite instability (MSI) due to loss of mismatch repair (MMR) proteins is associated with progression of MC, especially in the right colon.^4^ Second, although colorectal cancer (CRC) usually involves intramural growth, there have been several case reports of CRC with extramural progression, and the frequency of MC having this growth pattern is relatively high.^5,6^

Intestinal spirochetosis (IS) is a large bowel infection caused by the attachment of *Brachyspira aalborgi* and *B pilosicoli* to the colonic epithelium.^7^ It is suggested that a pathogenic mechanism of IS based on the destruction of colonic microvilli and colitis is histologically documented.^7^ It is also reported that IS may be responsible for colonic adenoma or hyperplastic polyps.^8^ Recently, a report revealed that IS must be associated with sessile serrated adenomas/polyps (SSA/P) that developed from MSI through the serrated pathway via methylation of the *MLH1* gene.^8–10^ It still remains unclear whether IS itself can be a pathogenic cause to develop CRC.

Here we report a case of MC with normal expressions of MMR proteins originating in the setting of chronic colitis associated with IS. This patient also had an intestinal stricture due to MC, making endoscopic diagnosis difficult. Hence, we suggest that IS might give rise to intestinal inflammation that has a potential to induce colitis-associated CRC. Moreover, it is necessary to consider the presence of malignant neoplasm within an intestinal stricture even if we suspect that the stricture is due to chronic inflammation, because some cases of MC have extramural progression and no obvious atypical colonic glands in biopsy specimens.

## CASE PRESENTATION

A 63-year-old Japanese man with no symptoms had a positive fecal occult blood test. His past medical history included a duodenal ulcer at 50 years of age.

**FIGURE 1:**
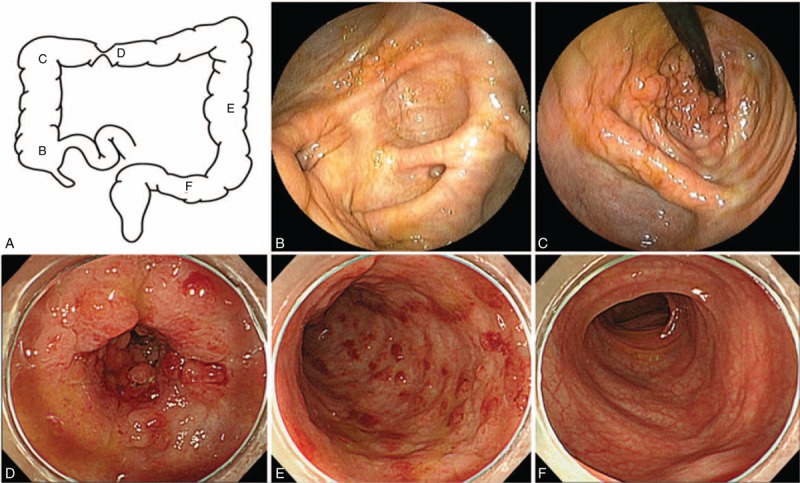
(A) Diagram of colon. (B) Representative endoscopic image of inflammatory scars in the cecum. (C) Representative endoscopic image of the intestinal stricture in the transverse colon (oral side) and inflammatory scars in the hepatic flexure. (D) Representative endoscopic image of the transverse colon (anal side) intestinal stricture caused by inflammatory polyps. (E) Representative endoscopic image of descending colon showing loss of the typical vascular pattern and haustra, indicating coarse, inflammed mucosa. (F) An endoscopic image of the proctosigmoid colon reveals normal mucosa.

**FIGURE 2:**
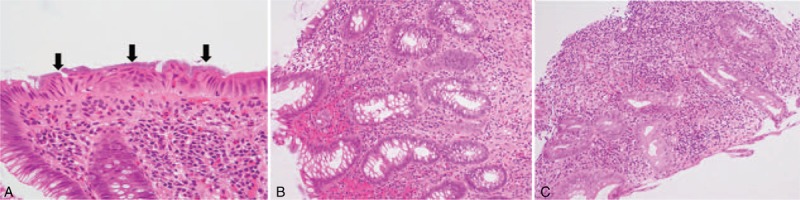
(A) Histopathological examination of colonic mucosa reveals basophilic fringes on the luminal surface of intestinal mucosa (black arrows) and infiltration of lymphocytes and eosinophils in a subepithelial lesion. (Hematoxylin and eosin stain, ×400). (B) At the initial evaluation, findings included colonic mucosa with infiltration of lymphocytes and eosinophils in a subepithelial lesion with no obvious dysplastic changes. (Hematoxylin and eosin stain, ×200). (C) Eight months after the initial evaluation, colonic mucosa contains an infiltration of lymphocytes and eosinophils with no obvious atypical colonic glands. (Hematoxylin and eosin stain, ×200).

**FIGURE 3:**
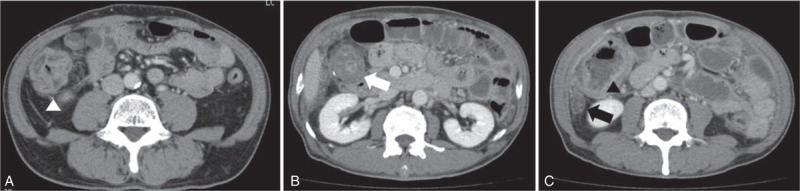
(A) Computed tomography (CT) of abdomen revealed wall thickening of the transverse colon around the hepatic flexure (white arrowhead) at the initial evaluation. (B) and (C) At 20 mo after the initial evaluation, abdominal CT revealed exacerbation of the intestinal stricture in the transverse colon around the hepatic flexure (B, white arrow), dilatation of the ascending colon (C, black arrowhead), and extraintestinal fluid accumulation, which indicated abscess formation (C, black arrow).

The colonoscopy revealed, in the observation range, many scars caused by inflammation in the cecum and ascending colon, an intestinal stricture caused by inflammatory polyps in the transverse colon (Figure 1A–D), loss of both visible vascular patterns and haustra in the descending colon (Figure 1E), and normal mucosa in the sigmoid colon (Figure 1F), rectum, and ileum. Examination of biopsies from each segment of colon (including even specimens from normal mucosa) revealed chronic colitis accompanied by IS, as evidenced by basophilic fringes on the luminal surface of the intestinal mucosa and infiltration of lymphocytes and eosinophils in subepithelial lesions (Figure 2A). There were no obvious dysplastic changes (Figure 2B). Abdominal computed tomography (CT) revealed wall thickening in the transverse colon around the hepatic flexure (Figure 3A). The upper endoscopy and enteroscopy findings were within normal limits. Laboratory data, including carcinoembryonic antigen, carbohydrate antigen 19-9, interferon-gamma release assay (IGRA), purified protein derivative skin test, *Entamoeba histolytica* antibody, human immunodeficiency virus antibody, and fecal examinations, including culture and polymerase chain reaction for *Mycobacterium tuberculosis*, revealed no remarkable findings. Hence, his condition was diagnosed as chronic colitis associated with IS. Metronidazole (MNZ) 1500 mg/d for 14 days was initiated to treat the IS. Although spirochetes were eliminated in pathological examinations, MNZ was not effective for the intestinal stricture of the transverse colon. We performed colonoscopy 4 times for 20 months since the fecal occult blood test was positive and each biopsy indicated colitis but no obvious malignancy (Figure 2C, Table 1).

**TABLE 1:**
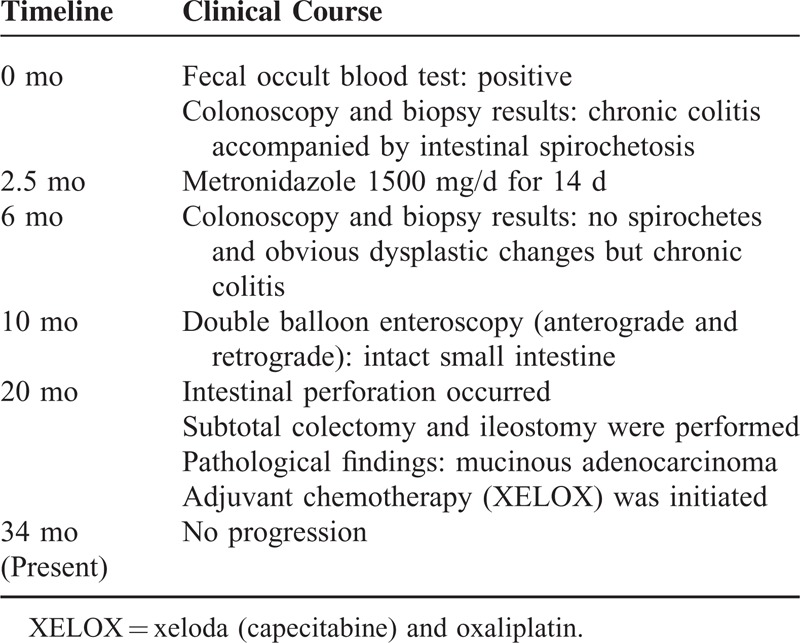
Timeline of Clinical Course

**FIGURE 4:**
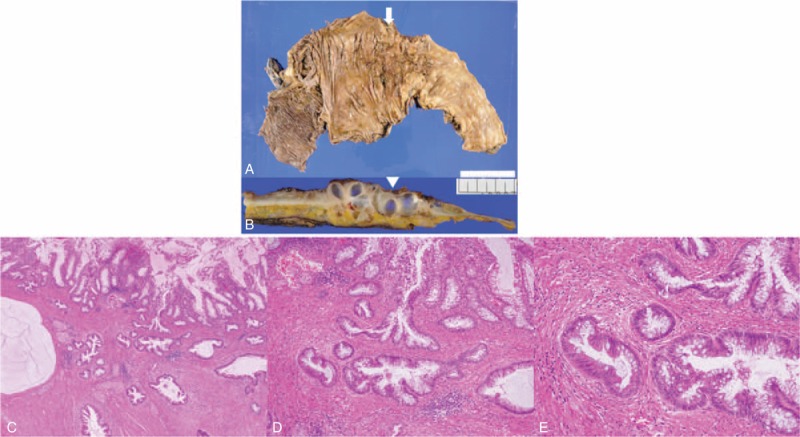
(A) Gross histology revealed circumferential coarse mucosa due to tumor formation in the transverse colon (white arrow). (B) On cross-section, the tumor was composed of cystic lesions that contained mucin (white arrowhead). (C)–(E) Histopathological examination revealed features of mucinous adenocarcinoma, including formations resembling lakes of mucin. Mucinous columnar epithelium glands with mild atypia infiltrated the muscularis propia and subserosal fatty tissue layers. (Hematoxylin and eosin stain – (C): ×40, (D): ×100, (E): ×200.)

**FIGURE 5:**
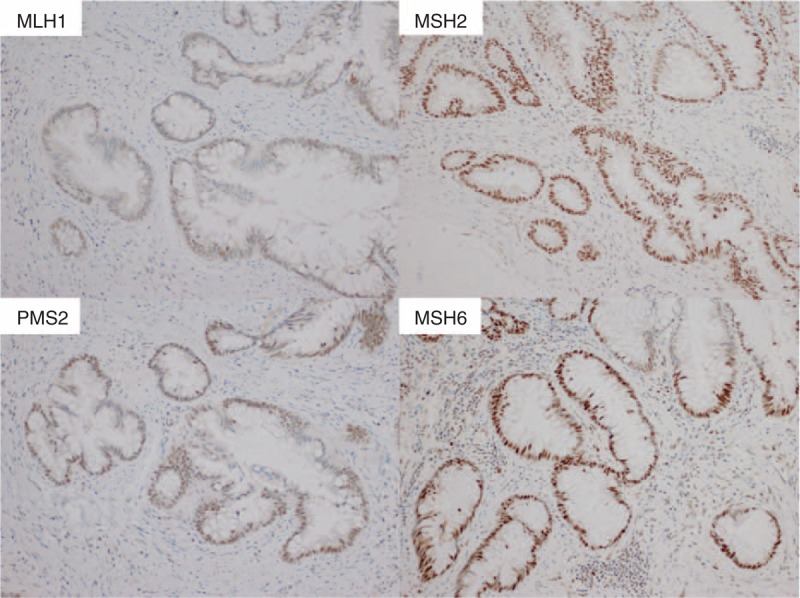
Immunohistochemical staining for DNA mismatch repair protein showed normal expression of MLH1, MLH2, PMS2, and MSH6. (×200).

The patient was admitted to our hospital with an acute abdomen 20 months after the initial presentation. He had urgent abdominal pain, constipation, and fever. Abdominal CT revealed exacerbation of the intestinal stricture in the transverse colon around the hepatic flexure (Figure 3B) and dilatation of the ascending colon with extraintestinal fluid (Figure 3C). We strongly suspected intestinal perforation due to obstruction. Subtotal colectomy and ileostomy were performed and the symptoms resolved. Histopathological examination of the specimens revealed MC that had infiltrated into the muscularis propia and subserous layers of the visceral peritoneum, purulent peritonitis due to perforation within the tumor and lymph node metastasis (Figure 4). The final histopathological diagnosis was a pT4a (invasion of serosa of peritoneum), pN1 (1/12), Ly0, V0, M0, and R0 tumor, corresponding to the Union for International Cancer Control IIIB classification. An immunohistochemical stain for DNA MMR proteins, including MLH1, MSH2, PMS2, and MSH6, revealed no lack of these expressions (Figure 5). Intensified adjuvant chemotherapy according to the Xeloda (capecitabine) and oxaliplatin schema was initiated. Currently, after 14 months of follow-up, the patient is without detectable tumor recurrence, as shown by abdominal CT scan and ultrasound examination (Table 1).

## DISCUSSION

We recognized 2 important clinical features of MC by treating the current case: MC can originate from colitis associated with IS, and diagnosing MC using endoscopic biopsy results can be difficult.

### Pathophysiology of MC Originating From Colitis Associated With IS

The etiological pathways of development of MC can be divided into the following 2 categories: intestinal inflammation, and MSI due to loss of MMR protein expression. In the current case, according to immunohistochemical staining results, expression of MMR proteins was normal (Figure 5). Therefore, MC seemed to originate from the colitis. Because of the pathological findings, the cause of colitis was thought to be IS (Figure 2A).

IS and its clinical significance has been debated for years. IS is characterized by end-on attachment of the spirochetes *B aalborgi* or *B pilosicoli* to the epithelial surface of colonic mucosa and resulting in the destruction of colonic microvilli and colitis with inflammatory infiltration associated with clinical symptoms such as chronic mucosal diarrhea, rectal bleeding, abdominal pain, and weight loss.^7,11^ In a report of 11 cases of IS, most patients showed mild nonspecific colonic inflammation in pathological findings.^12^ As endoscopic findings, nonspecific ulceration of ileocecal valve and ischemic ulcers with thickened folds in the entire colon were observed in 2 patients.^12^ In the current case, spirochete was only a pathogen that induced nonspecific colitis.

Delladetsima et al^13^ claimed that human IS is frequently associated with various intestinal diseases, including carcinoma, adenomatous polyp, metastatic polyp, and ulcerative colitis. Colonic carcinoma was the most frequently associated condition, because chronic stasis of intestinal contents favors infestation by the spirochetes.^8,13^ Furthermore, a recent report indicated an association between IS and SSA/P, which can develop in the setting of MSI.^9^ In addition, SSA/P can be considered to be a precursor to some types of MSI-high carcinomas of the proximal colon, such as MC.^14,15^

Hence, we hypothesize that the sequence of MC development in this case was as follows: IS→SSA/P→MC. However, we verified normal expression of MMR proteins, implying microsatellite stability, and therefore propose an alternative sequence as follows: IS→colitis→MC. Repeated tissue damage and regeneration due to chronic inflammation lead to the presence of highly reactive nitrogen and oxygen species released from inflammatory cells, which interact with DNA in proliferating epithelium, resulting in permanent genomic alterations such as point mutations, deletions, or rearrangements.^16^ Hussain et al^17^ hypothesized that the high frequency of the *p53*-mutated allele in nondysplastic mucosa of chronic colitis may confer susceptibility to the development of CRC in an inflammatory microenvironment. Although a pathophysiological association between neoplasms and IS, especially whether spirochetes themselves can be direct pathogens to develop MC, remains to be demonstrated, the intestinal inflammation due to IS might induce genomic alterations related to MC development.

### Clinicopathological Characteristics of MC That Render Making the Correct Diagnosis of MC Difficult

The growth pattern of CRC is generally intramural progression. On the other hand, extramural progression of CRC can occasionally occur. Although the MC frequency in total cases of CRC is 2.9%, the MC percentage among cases of CRC with extramural progression is 26.2%.^5,18^ Therefore, MC is a major pathological type of CRC with extramural progression. In the current case, glands consisting of mucinous columnar epithelium with low-grade atypia infiltrated into the muscularis propia and produced mucus. Through this mechanism, extramural progression could occur. Moreover, the formation of an intestinal stricture would be relevant to the expansion of the large amount of mucus that the atypical MC glands produce in the deep layers (Figure 4A–C). Hence, it may not be possible to visualize obvious dysplasia of the intestinal stricture prior to surgery.

Meanwhile, there is the question whether colonic glands in preoperative endoscopic biopsy specimens that appeared to be normal could have been recognized as low-grade atypia (especially Figure 2C). In reality, pathological discrimination between normal glands and atypia, including low grade, is difficult before surgery; the endoscopic biopsy included colonic glands without obvious cellular and structural atypia (Figure 2B and C). After examination of the resected specimens, the atypia of the colonic glands was also found to be mild (Figure 4C–E). On the contrary, our case suggests that, even when the endoscopic biopsy reveals no obvious atypical glands, MC should not be ruled out, especially when there is an intestinal stricture, because colonic glands appearing normal might actually have dysplastic findings of MC.

Furthermore, we realized that the endoscopic findings of colitis distracted us from making the correct diagnosis of MC. At first, we believed that the intestinal stricture could be formed by the colitis, including IS or IBD, and CRC did not come to mind as a priority in the differential diagnosis. This case suggests that we should perform a boring biopsy or endoscopic ultrasound (EUS) to clarify the cause of intestinal stricture, even if we suspect it is due to chronic inflammation, because of the unique characteristics of MC discussed above. Actually, Shimizu et al^19^ previously suggested that EUS is a helpful method for detecting invasive cancer, including MC, associated with ulcerative colitis.

In conclusion, we treated a case of MC arising in the setting of chronic colitis associated with IS. This report discusses the possibility of an association between IS, as bacterial infection, and neoplasm formation. The inflammation, rather than MSI, might be more relevant to the mechanism of tumor development. Furthermore, MC in the current case had extramural progression, no obvious atypical glands in endoscopic biopsy specimens, and was accompanied by intestinal inflammation. We suggest that MC can be overlooked because of these unique clinicopathological features, and that clinicians should keep this in mind when treating patients having a similar presentation.
